# An eXplainability Artificial Intelligence approach to brain connectivity in Alzheimer's disease

**DOI:** 10.3389/fnagi.2023.1238065

**Published:** 2023-08-31

**Authors:** Nicola Amoroso, Silvano Quarto, Marianna La Rocca, Sabina Tangaro, Alfonso Monaco, Roberto Bellotti

**Affiliations:** ^1^Dipartimento di Farmacia-Scienze del Farmaco, Universitá degli Studi di Bari Aldo Moro, Bari, Italy; ^2^Istituto Nazionale di Fisica Nucleare, Sezione di Bari, Bari, Italy; ^3^Dipartimento Interateneo di Fisica, Universitá degli Studi di Bari Aldo Moro, Bari, Italy; ^4^Dipartimento di Scienze del Suolo, della Pianta e degli Alimenti, Universitá degli Studi di Bari Aldo Moro, Bari, Italy

**Keywords:** Alzheimer's disease, XAI, brain connectivity, explainability, MCI

## Abstract

The advent of eXplainable Artificial Intelligence (XAI) has revolutionized the way human experts, especially from non-computational domains, approach artificial intelligence; this is particularly true for clinical applications where the transparency of the results is often compromised by the algorithmic complexity. Here, we investigate how Alzheimer's disease (AD) affects brain connectivity within a cohort of 432 subjects whose T1 brain Magnetic Resonance Imaging data (MRI) were acquired within the Alzheimer's Disease Neuroimaging Initiative (ADNI). In particular, the cohort included 92 patients with AD, 126 normal controls (NC) and 214 subjects with mild cognitive impairment (MCI). We show how graph theory-based models can accurately distinguish these clinical conditions and how Shapley values, borrowed from game theory, can be adopted to make these models intelligible and easy to interpret. Explainability analyses outline the role played by regions like putamen, middle and superior temporal gyrus; from a class-related perspective, it is possible to outline specific regions, such as hippocampus and amygdala for AD and posterior cingulate and precuneus for MCI. The approach is general and could be adopted to outline how brain connectivity affects specific brain regions.

## 1. Introduction

Alzheimer's disease (AD) is a progressive neurodegenerative disease, which represents the seventh leading cause of mortality in the United States after when COVID-19 appeared at the top of this ranking (Alzheimer's Association, 2022). According to the World Health Organization (https://www.who.int/news-room/fact-sheets/detail/dementia), AD is the most common form of dementia (60–70%) which affects 55 million people all over the world and some studies estimated that over 150 million people will develop dementia by 2050 (Nichols et al., 2022). Alzheimer's disease involves the loss of neuronal connections, thus resulting in a connectivity damage that impairs neuronal functionality and eventually leads to their death. Neuronal death has macroscopic effects on the brain; specific brain regions start shrinking, this is what is usually known as brain atrophy (Devanand et al., 2007; Liu et al., 2019; Talwar et al., 2021).

From a clinical point of view, this feature could be highly beneficial because it can be revealed by imaging, especially by brain Magnetic Resonance Imaging (MRI) (Lerch et al., 2008; Vemuri et al., 2008; Julkunen et al., 2010). The relationships between the structure of the brain and its functional abilities can be investigated (Emre et al., 2007; Solé-Padullés et al., 2009; Frisoni et al., 2010) and, therefore, on the one hand it is possible to assess to which extent the disease severity reflects the structural damage, on the other hand it is possible to develop accurate diagnostic approaches based on the clinical symptoms. To this aim, it becomes extremely important to develop accurate diagnosis support systems which can detect early signs of atrophy, before symptoms appear, and to initiate timely treatments (Breijyeh and Karaman, 2020). In the last decades, several studies have investigated the structural changes in the brain and tried to correlate them to different stages of the disease severity, including preclinical AD, mild cognitive impairment (MCI), and clinically diagnosed AD (Sperling et al., 2011; Alzheimer's Association, 2020).

MCI condition is particularly interesting because its symptoms are not fully evident and brain damage is not extensive. MCI is characterized by memory loss episodes, difficulty thinking, and the first signs of physical problems (Gauthier et al., 2006; Petersen et al., 2014). In addition, not all MCI subjects convert to AD: according to estimates, 8 out of 10 people with MCI develop AD within 7 years, whereas there are patients which continue to have MCI or convert back to the normal condition years later (Larrieu et al., 2002; National Institute on Aging, 2002; Gauthier et al., 2006; Tábuas-Pereira et al., 2016). It would therefore open up a wide range of possibilities for cures and disease-modifying therapies in the event we could effectively diagnose MCI and distinguish physiological impairment from early symptoms of AD (Huckans et al., 2013; Huang et al., 2020).

Neuroimaging studies have demonstrated their effectiveness to investigate brain changes and identify the first signs of disease (Lebedeva et al., 2017; Zeng et al., 2021). In particular, some studies have investigated the use of graph theory (Bullmore and Sporns, 2009; de Haan et al., 2012; Tijms et al., 2013), the conversion from MCI to AD and algorithms for studying AD (Daianu et al., 2015; Teipel et al., 2016; Liu et al., 2017). During the last several decades, machine learning techniques have demonstrated their ability to perform binary and multi-label classification tasks (Gupta et al., 2019; Kim et al., 2021; Sheng et al., 2021; Song et al., 2021); this is especially important when dealing with MCI, which intrinsically represents a heterogeneous clinical condition, often presenting both normal and pathological behavior. However, clinical practitioners have difficulty adopting these techniques due to the fact that they are often considered as black boxes, difficult to interpret.

In this work, we employ a brain connectivity model based on “patches”, whose effectiveness has already been verified for AD classification (La Rocca et al., 2017, 2018; Amoroso et al., 2018b,c, 2019). This overcomes the typical issues of voxelwise and region-based approaches: (i) it removes the computational burden and overfitting concerns associated with voxelwise methods (Goenka and Tiwari, 2022), but also the parametric statistical methods turned out to be over-conservative for voxelwise inference (Eklund et al., 2016; Górriz et al., 2021); (ii) using unsupervised segmentation of the brain, this approach does not require region of interest (ROI) localization based on prior biological knowledge to extract regional features, and it provides a better way to detect microscopic structural changes in the brain that ROI extracted features cannot capture (Amoroso et al., 2015).

Here, we take a step beyond by investigating to which extent such model can “explain” the effects of AD on brain connectivity: to this aim, we consider a consolidated eXplainable Artificial Intelligence (XAI) approach based on Shapley values (Messalas et al., 2019; Loh et al., 2022). The use of XAI methods to characterize neurodegenerative diseases and, more in general, to equip neuroimaging studies is rapidly increasing (El-Sappagh et al., 2021; Anjomshoae and Pudas, 2022; Lombardi et al., 2022). A lot of neuroimaging fields have seen a highly increased interest in the application of XAI techniques, obtaining benefits from transparency provided by these approaches (Farahani et al., 2022). MRI research is exploiting these techniques to study brain aging both through *ante-hoc* interpretability models, such as stability assessment or latent variable models, and post hoc models, such as feature importance and saliency maps (Galazzo et al., 2022; Qian et al., 2023). XAI techniques were used to study the conversion from MCI to AD by high-density electroencephalography (HD-EEG) to detect which EEG-channels and range of frequencies were most predominant in disease progression (Morabito et al., 2023). In addition, clinical cognitive tests are getting advantage from the application of XAI, which provides insights into the cognitive processes by visualizing and identifying the specific cognitive features that are most influential in determining the test outcomes; using a single test such as the Clock Drawing Test or selecting a subset of cognitive tests to exploit XAI scores for individualized prediction explanations (Beebe-Wang et al., 2021; Jimenez-Mesa et al., 2023).

We initially demonstrate to which extent the patch-based approach is reliable for characterizing patients, controls and MCI subjects. Then, the classification performance and its reliability are investigated in order to ensure the model is sound. Finally, an overall explanation of the model and an explanation of its decision is provided by means of Shapley values.

## 2. Materials and methods

### 2.1. Imaging the brain and building a network model

In this research, we used a dataset composed of 432 brain T1 MRI images, relative to 126 normal control (NC), 214 MCI and 92 AD subjects from the Alzheimer's Disease Neuroimaging Initiative (ADNI). ADNI is a longitudinal multicenter study designed to obtain early diagnosis and monitoring of AD through the study and development of clinical, imaging, biochemical, and genetic biomarkers (https://adni.loni.usc.edu/about/). ADNI images were normalized using the MNI152 brain template with 197 × 233 × 189 mm^3^ size and 1 × 1 × 1 mm^3^ resolution; from now onward voxel and 1 mm^3^ will be interchangeably used. In the following Table 1 the number of instances, gender information, age, years of education, and Mini Mental State Examination (MMSE) score are enlisted. This cohort of MRI scans passed quality control (QC) using the Laboratory of Neuro Imaging (LONI) QC System (Kim et al., 2019) so that only high quality images were considered without signal alterations.

**Table 1:** Demographic information for each class.

	**AD**	**MCI**	**NC**	**Total**
Number of instances	92	214	126	432
Female/male	43/49	86/128	65/61	194/238
Age (years)	75.82 ± 7.60	75.30 ± 7.13	75.61 ± 5.58	75.50 ± 6.81
Education (years)	15.10 ± 3.26	15.52 ± 3.30	16.05 ± 2.61	15.58 ± 3.11
MMSE	23.45 ± 1.95	26.98 ± 1.75	29.16 ± 0.98	26.86 ± 2.59

The table provides the number of instances, gender information, and also age, years of education MMSE, with mean and standard deviation.

**Figure 1:**
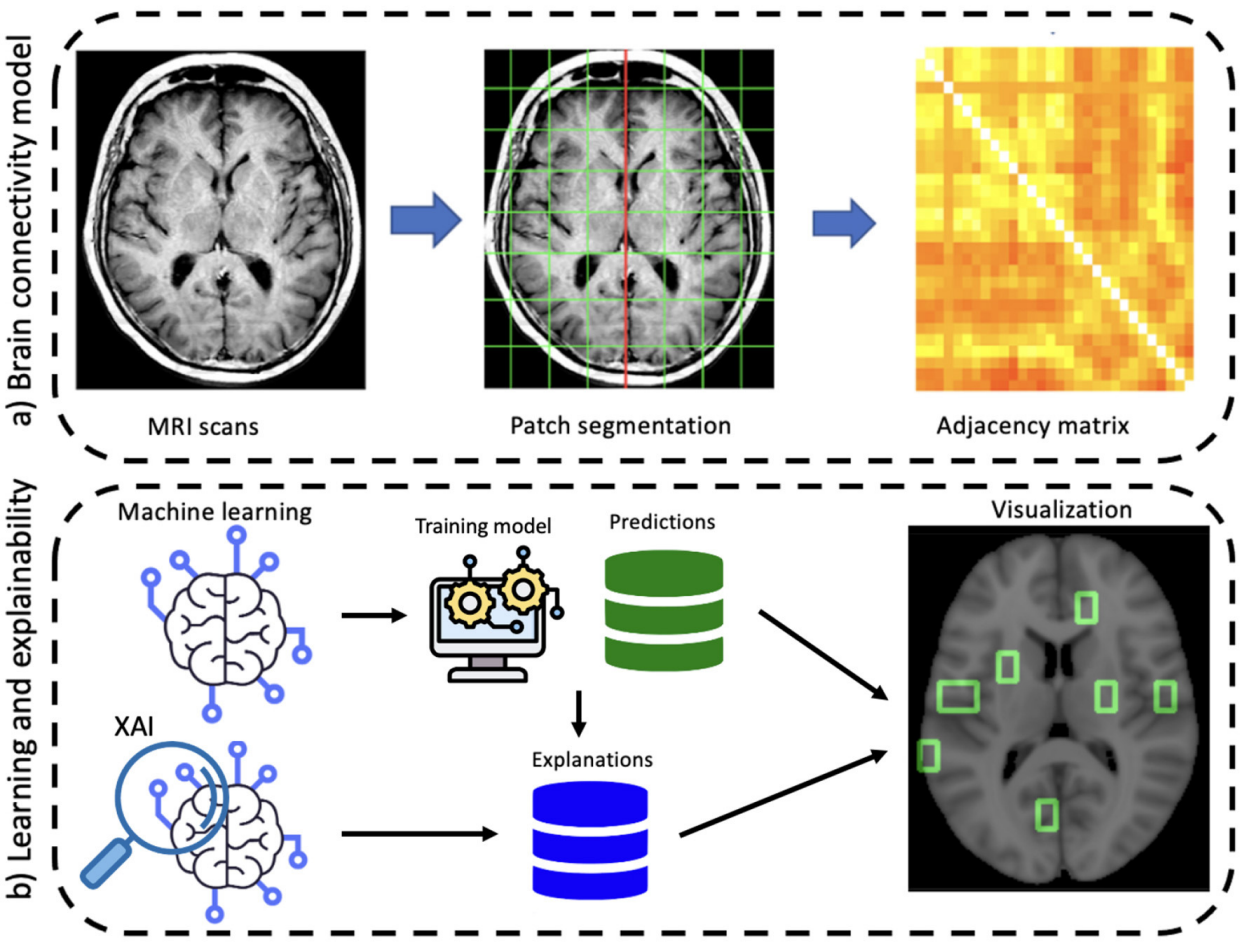
A schematic overview of the proposed workflow divided into its main two phases: **(A)** brain connectivity model and **(B)** explainable machine learning.

The proposed approach involves two main stages: the construction of the network model and the learning and explainability phase (see Figure 1). First, a graph theory-based model is employed to study structural brain connectivity and identify early signs of Alzheimer's disease. Accordingly, each patient's brain is modeled as a complex network. Brain MRIs are parceled into rectangular boxes called “patches” of fixed dimensions that represent the network's nodes; the links are obtained in terms of nodes' pairwise similarity measured by absolute Pearson's correlation. As a difference with previous works where multiplex networks were adopted, here we consider single-subject networks to achieve a simpler description and, therefore, a more interpretable model. In fact, using single-subject networks, network features can be directly related to single nodes and then to specific brain regions.

Image processing was the first algorithmic step. Using Oxford FMRIB software library (FSL) (Jenkinson et al., 2012), images were skull-stripped and spatially normalized as well in intensity to mitigate data heterogeneity, an aspect of fundamental importance for studying a multi-center database such as the one provided by ADNI. First bias field correction and skull stripping was performed using FSL Brain Extraction Tool (BET) (Smith, 2002). Thereafter, spatial normalization was performed to ensure co-registration to the MNI152 template using FSL Linear Registration Tool (FLIRT) (Jenkinson et al., 2002); in particular, an affine registration was adopted with default parameters.

**Figure 2:**
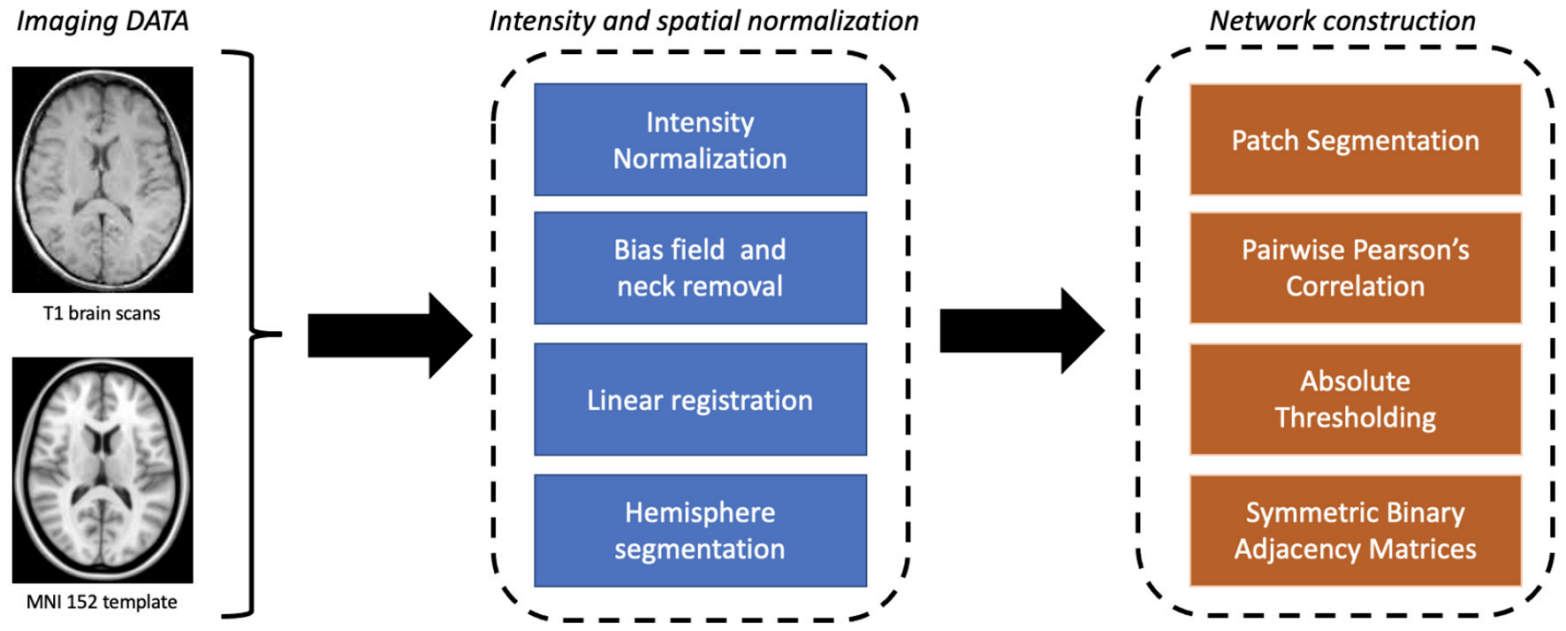
A detailed flowchart from imaging data to network modeling.

After registration, by using the medial longitudinal fissure, normalized brains were divided into the two hemispheres; each hemisphere was then covered by an equal number of patches of fixed dimensions *l*_1_×*l*_2_×*l*_3_ (patches overlapping the template with < 10% of voxels were neglected). For the patch dimension, we considered the 10 × 15 × 20 mm^3^ configuration, corresponding to a total volume of 3, 000 mm^3^, for a total of 549 patches (see Figure 2).

Accordingly, we built an undirected weighted network for each MRI scan with 549 nodes. Pearson's correlation coefficient was chosen for links for three main reasons: (i) it has an affordable computational cost; (ii) it is easy to implement, and (iii) it easy interpret in terms of brain atrophy. Absolute values were considered to take into account left/right symmetry of the brain; finally, a threshold was used to remove weak correlations (< 0.3) that could raise noisy connections.

Once structural connectivity had been modeled in terms of a complex network, we exploited such characterization to evaluate some network metrics and outline the effects of cerebral atrophy on brain connectivity. Furthermore, since network metrics can be easily divided in local and global ones, to acquire a detailed description of how AD differently affects distinct anatomical districts, nodal centrality measures were preferred. In particular, three graph metrics were used: strength, betweenness, and eigenvector centrality. The strength of a node is a local centrality measure defined by the sum of edge weights of each node; as a difference, a global centrality measure such as eigenvector centrality takes into account also the influence of a node in a network based on the amount of nodes with high number of connections which it is connected to. Finally, a dynamic centrality measure is considered: the betweenness; betweenness is the ratio between the shortest paths connecting two nodes passing through a specific node and all available shortest paths. Accordingly, it is a measure capturing the information flow within a network (Amoroso et al., 2021; Bellantuono et al., 2021; Sheng et al., 2021).

After calculating these three metrics for each node of the adjacency matrices, a matrix representation *M*×*n* of the data was obtained, where *M* is the number of enrolled patients and *n* is the total number of features (number of metrics used times number of nodes). Here, the resulting matrix had dimensions of 432 × 1, 647.

### 2.2. Learning AD patterns

We have used the previously defined matrix representation to train a machine learning model. The main objectives we set during this learning phase were to achieve a sound representation of AD and verify the robustness and reliability of the connectivity network model. To this aim, a three-label classification was performed for AD, MCI, and NC subjects. All these analyses were carried out within a 10-fold cross-validation framework; the procedure was iterated 50 times to estimate performances and uncertainties; besides, to ensure balanced cross-validation splits between training and test, stratified sampling was used.

To ensure that the observed performance was due to the informative content provided by the network features independently from the classification model used, we compared the performance of several classifiers: Random Forest (RF) (Breiman, 2001), Support Vector machine (SVM) (Cortes and Vapnik, 1995), eXtreme Gradient Boosting (XGBoost) (Chen et al., 2015), Naive Bayes (NB) (Rish et al., 2001), and Logistic Regression (LR) (Ng and Jordan, 2001) were used.

### 2.3. From classification to explanations

After classification performance accuracy and reliability had been assessed, explainability analyses were carried out by means of SHapley Additive exPlanations (SHAP) (Lundberg and Lee, 2017). Accordingly, it is possible to evaluate to which extent each feature affects the model's predictions. By averaging the impact of a feature on the whole dataset, a feature importance map can be achieved; at the same time, this explanation allows for the identification of the factors which determine the classifier's decisions for each subject. Also, this analysis provides additional information compared with standard feature importance approaches in that it does not determine the importance of one feature but also it explains how its value, combined with the other features' values, leads to the decision taken by the algorithm. Moreover, it is possible to compare whether the impact of features on a single decision is coherent with the overall feature importance (the one related to the three-class classification model) or not. Finally, an important aspect to remark is that SHAP can be adopted with different machine learning algorithms (Strumbelj and Kononenko, 2010), thus making the proposed approach algorithm-independent.

A fundamental aspect of SHAP concerns their computational burden, as the computational load increases exponentially with the number of features. To overcome this issue, a specific experimental design is proposed. Explainability analyses were nested in the repeated 10-fold cross-validation. Using a RF classifier, for each cross-validation round a subset of important features was selected by means of the combined use of Mean Decrease Accuracy (MDA) and statistical significance.

This procedure can be described in the following two steps: (i) firstly, the features exceeding the 95th percentile of the MDA distribution were selected within each cross-validation round; (ii) then, these newly selected features were further reduced considering statistical significance: only the features with the 5% significance using the one-sided binomial test were kept. On average, the number of features selected during each cross-validation round was ~50. During each cross-validation round, this final set of important features was used to train a second classification model on the same training set with reduced number of features, in order to evaluate its informative content and to carry out the explainability analyses. Shapley values were calculated for each of the test subjects of each cross-validation round. The same analysis was carried out for each available class by considering three one-vs.-all classifiers to outline the presence of class-specific behaviors (Whitwell et al., 2007; Byun et al., 2015; Cabral et al., 2015). The mentioned analyses were carried out with R, with the *DALEX* and *shapviz* packages (Biecek, 2018; Biecek and Burzykowski, 2020; Michael Mayer, 2022). Finally, to validate the results from a clinical perspective, we determined which specific brain regions were associated with the selected important features (as each feature was directly related to a patch). To this aim, the Talairach atlas (Lancaster et al., 1997, 2000) was used.

## 3. Results

### 3.1. Robustness and reliability

**Figure 3:**
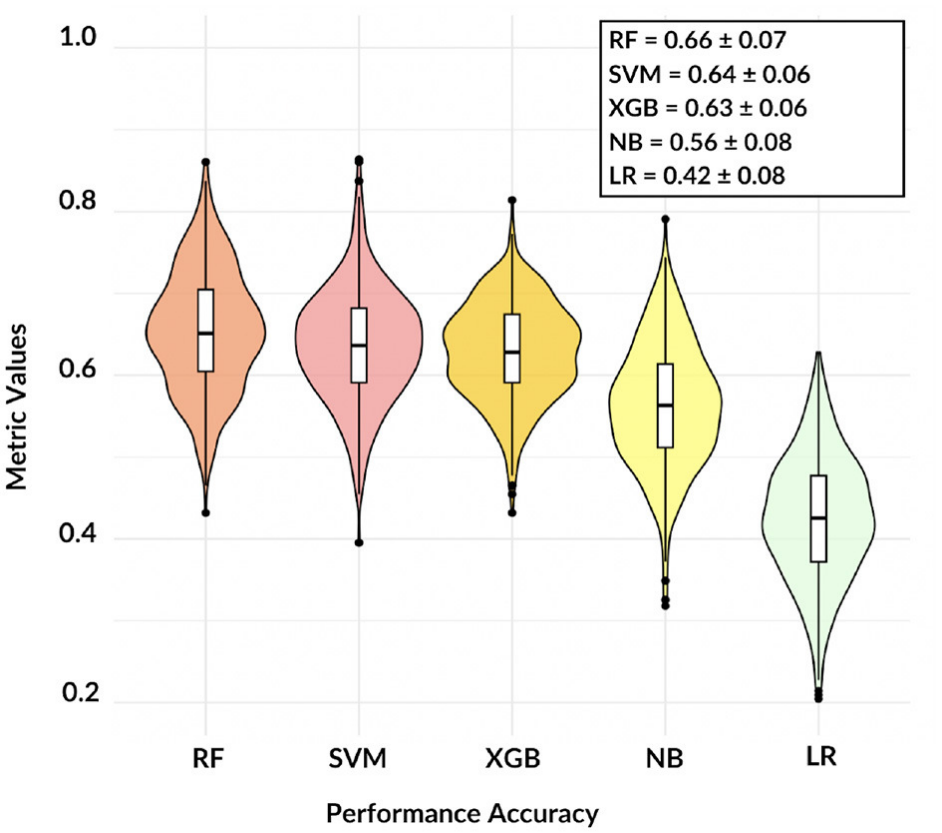
The violin plot shows the comparison among five different models, except NB and LR classification remains accurate over 60%.

Firstly, we investigated the validity and reliability of the brain connectivity model. To this aim, a 10-fold cross-validation analysis was repeated 50 times, results are shown in Figure 3.

These findings demonstrate that, except for LR, the adopted models are consistent and the informative power of the network features can reach satisfactory values: in terms of accuracy, RF resulted the best model with a mean three-class accuracy of 0.66 ± 0.07.

**Figure 4:**
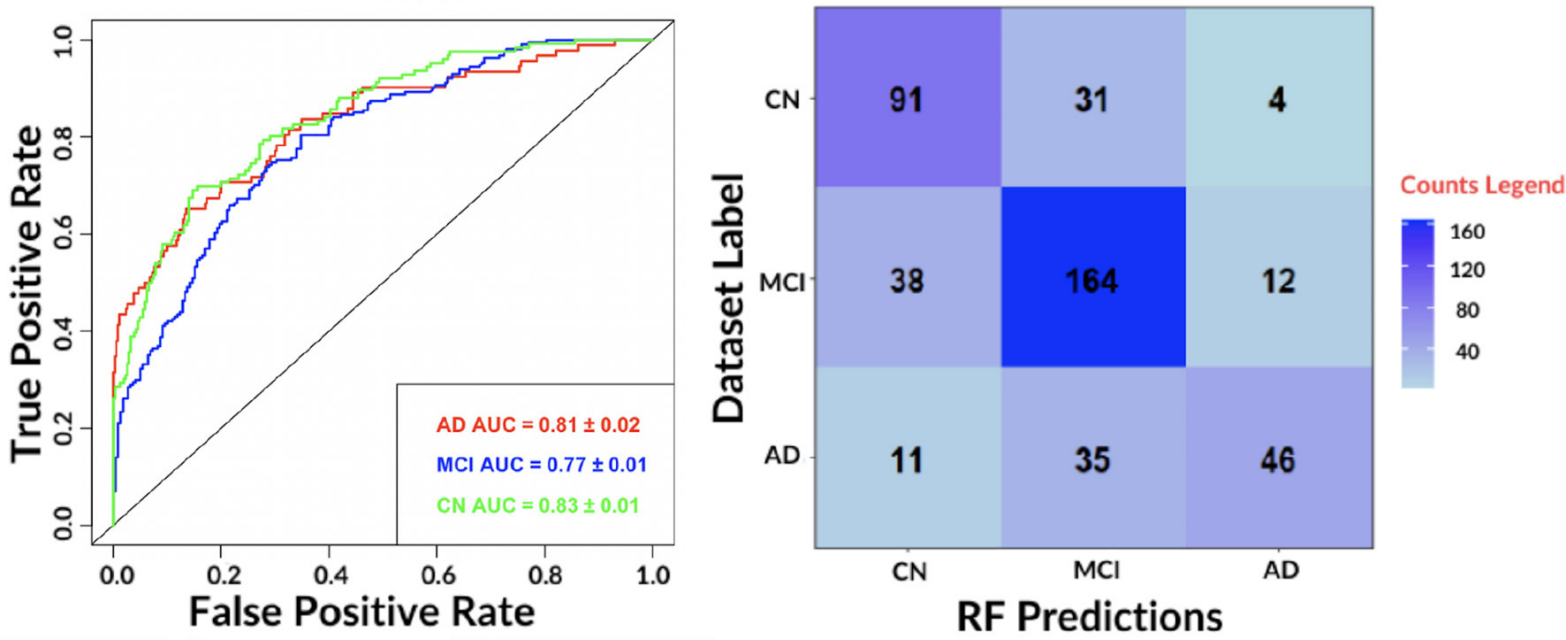
**(Left)** The receiver-operating-characteristic (ROC) curve on all test data for the best performing algorithm: RF. **(Right)** The confusion matrix for the RF.

To obtain class-specific evaluations, ROC curves and their area under the curve (AUC) were taken into account (see Figure 4).

The results show an accurate classification for all classes: 0.81 ± 0.02 for AD, 0.77 ± 0.01 for MCI and 0.83 ± 0.01 for NC. The contingency table allows to appreciate how MCI and AD include most of misclassifications, 50 and 46, respectively. We also evaluated the agreement of the predictions of the best performing classifiers: RF and SVM by means of Pearson's correlation. We found a 0.79 correlation for AD, 0.70 for MCI and 0.78 for NC. Hence, for subsequent analyses, only the RF model was considered.

### 3.2. Characterizing AD, MCI, and NC patterns

**Figure 5:**
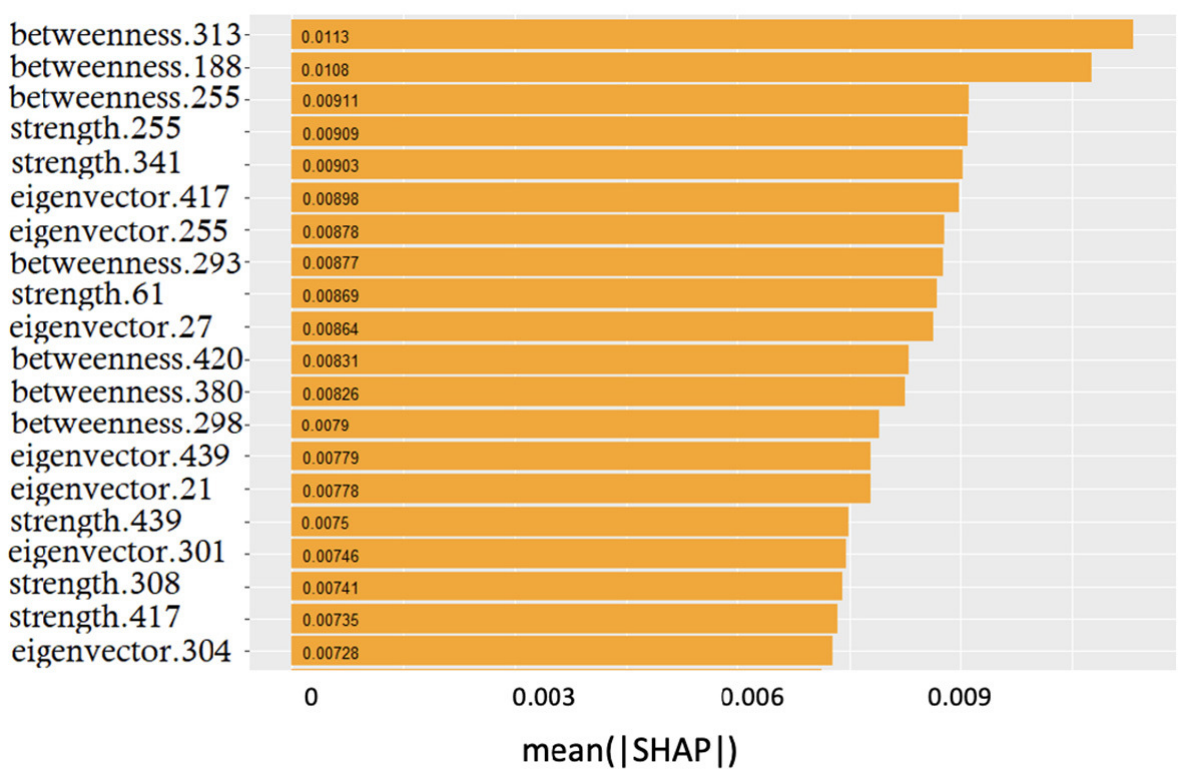
Global feature importance expressed in terms of mean absolute SHAP values: the metric and the related patch are indicated.

Once demonstrated the reliability of the base of knowledge, we investigated whether the features driving classification remained the same despite class-specific differences. In Figure 5, it is possible to observe the overall (related to all three classes) ranking of features; only the top 20 features are shown for display purposes.

The smooth trend demonstrates that it is difficult to find few features dominating over the others. Interestingly, the top three positions were occupied by betweenness; however, the significant presence of both strength and eigenvector centrality features suggested that no metrics prevailed over the others.

Using the three class-specific models, three importance rankings were also constructed; besides, to outline class-related patterns, explainability analyses were carried out using only correctly classified subjects. These class-rankings were compared with the overall ranking by means of the Spearman's correlation coefficient ρ. The results are presented in Table 2.

**Table 2:** Agreement between the overall feature importance and the ones retrieved considering the three classes separately.

**Overall feature importance vs**	**Spearman's ρ**	***p*-value**
AD feature importance	0.06	0.6
MCI feature importance	0.73	< 2.2 × 10^−16^
NC feature importance	0.59	4 × 10^−8^

While MCI and NC rankings were significantly correlated to the overall ranking, the AD ranking showed a very weak correlation. These findings allow us to highlight how much brain connectivity is influenced by brain atrophy; particularly in AD patients, in which the high heterogeneity is more impactful than in subjects of the other two clinical classes.

Lastly, we analyzed the correlation among the three feature importance rankings restricted to only correctly classified subjects (see Table 3).

**Table 3:** Agreement between the feature rankings restricted to correctly classified subjects.

**Feature importance between classes**	**Spearman's ρ**	***p*-value**
AD vs. MCI	−0.23	0.04
AD vs. NC	−0.22	0.04
MCI vs. NC	0.08	0.46

The degree of correlation between these rankings allows us to understand to what extent the correct classification of subjects of different classes may depend on the order of importance of the features. Using Spearman's correlation, it is possible to determine the strength and direction of the monotonic relationship between two ranks. Although the observed correlations are definitely lower than the previous ones, at 5% significance anti-correlation between AD vs. MCI and AD vs. NC is detected. These negative Spearman correlation coefficients highlight a decreasing monotonic trend among the ranks of the features that better correctly classify the AD class compared to those of the other two clinical classes. These correlations, along with the previous ones, suggest the presence of coherent and distinguishable patterns for NC and MCI, whereas a more heterogeneous and elusive condition characterizes the AD class.

### 3.3. Explaining the observed patterns

SHAP were also used to examine the effects of features on predictions: how and how much each feature affected the prediction score.

**Figure 6:**
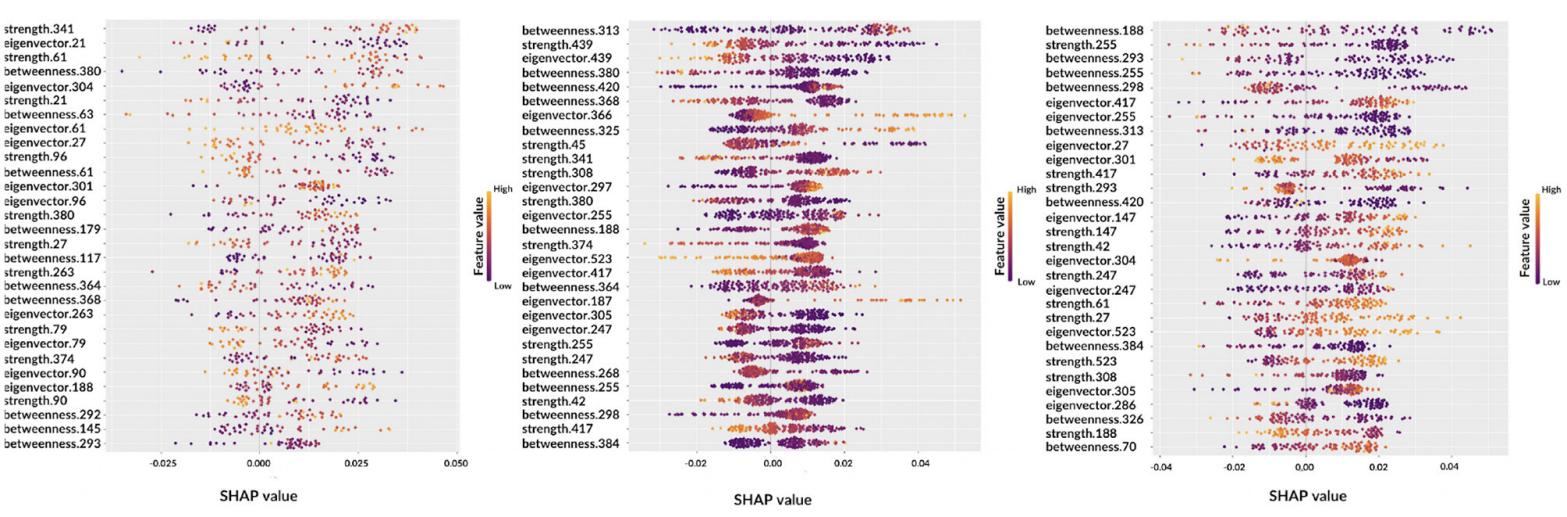
Beeswarm plots representing global feature explainability. From left to right, the graphs correspond to AD, MCI, NC.

This analysis provided additional information compared to the simple rankings showed in the previous section: the features are ranked from top to bottom, from the most important to the least important, and they are distributed based on both their impact on the prediction as well as their value, for high values, colors tend toward yellow, and for low values, colors tend toward purple. A positive Shapley value represents a positive impact on the correct predicted outcome, and a negative value implies a negative impact (see Figure 6).

Observing the beeswarm plots, the features in common between the three cases are of particular interest. In particular, for AD and MCI the betweenness of patches 364, 368, and 380 were found along with the strength of patches 374 and 380. Analogously, for AD and NC, strength of patches 27 and 61, eigenvector centrality of patches 27, 301, and 304 and betweenness of patch 293 were highlighted. Finally, MCI and NC showed in common: betweenness of patches 188, 255, 298, 313, 384, and 420; eigenvector centrality of patches 247, 255, 305, 417, and 523; strength of patches 42, 247, 255, 308, and 417. Notably, in some cases, the same patch was detected from different metrics.

**Figure 7:**
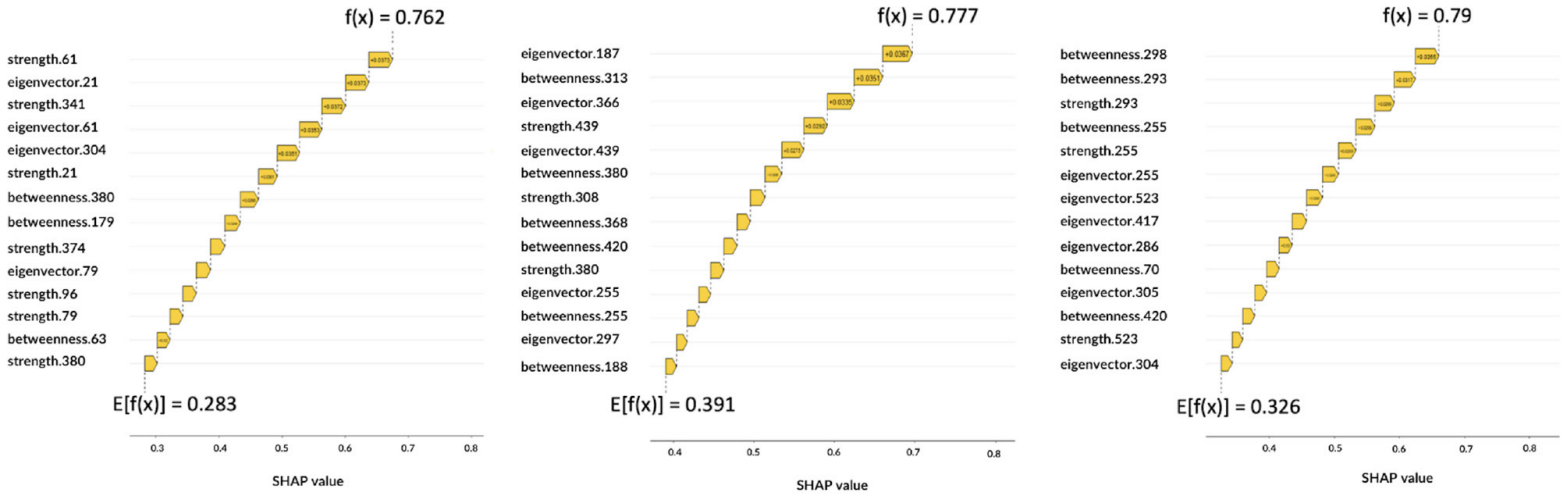
Local explanations: for each subject the impact of features on the decision is shown. From left to right, the graphs correspond to AD, MCI, NC.

Let us consider some specific cases. For AD and MCI classes, it is possible to observe the opposite behaviors of betweenness of patches 364 and 380. Thus, these features are important for the correct classification of AD subjects if their values are high; conversely, low values of these features are significant for the correct classification of MCI subjects. Similar findings can be obtained for other classes, for example, NC subjects are characterized by low values of eigenvector centrality of patch 27 while AD subjects tend to show are higher values. Interestingly, the importance of a feature for classification can differ from one class to another, for example the betweenness of patch 293 ranks third for NC subjects, but it is ranked last for AD patients. Understanding this findings without the help of anatomical interpretation can be extremely complicated, this is why in the following section the XAI visualization is presented. Another aspect which deserves to be outlined is the possibility to carry out personalized XAI analyses, which explain for each patient how classification was determined (see Figure 7).

The Figure 7 shows three correctly classified subjects belonging to AD, MCI, and NC, respectively, which correspond to three example cases to show possible differences with global XAI analysis. These graphs are waterfall plots and provide an analysis tool to visualize how each feature impacts on the average of the model output over the training data (baseline value E[f(x)]) for a specific subject. The bars represent the Shapley values of the features that most influentially drive the individual prediction. As can be seen from the three plots, positive values indicate a greater impact on the prediction, increasing the baseline predicted score. Longer bars indicate a greater influence of the feature and adding them together we obtain the final prediction f(x); the Figure 7 shows how this value has increased the initial baseline value. It is apparent from these graphs that some features extremely important from a global perspective (e.g., eigenvector centrality of patch 27 or betweenness of patch 61) and that are essential to the global analysis of classes, do not appear in the class-specific analyses. While global XAI explains on average which features are important for classification, subject-level XAI is of fundamental importance to clinically validate what happens for a specific patient; in anticipation of future developments toward personalized diagnostics.

### 3.4. From network metrics to brain regions

**Figure 8:**
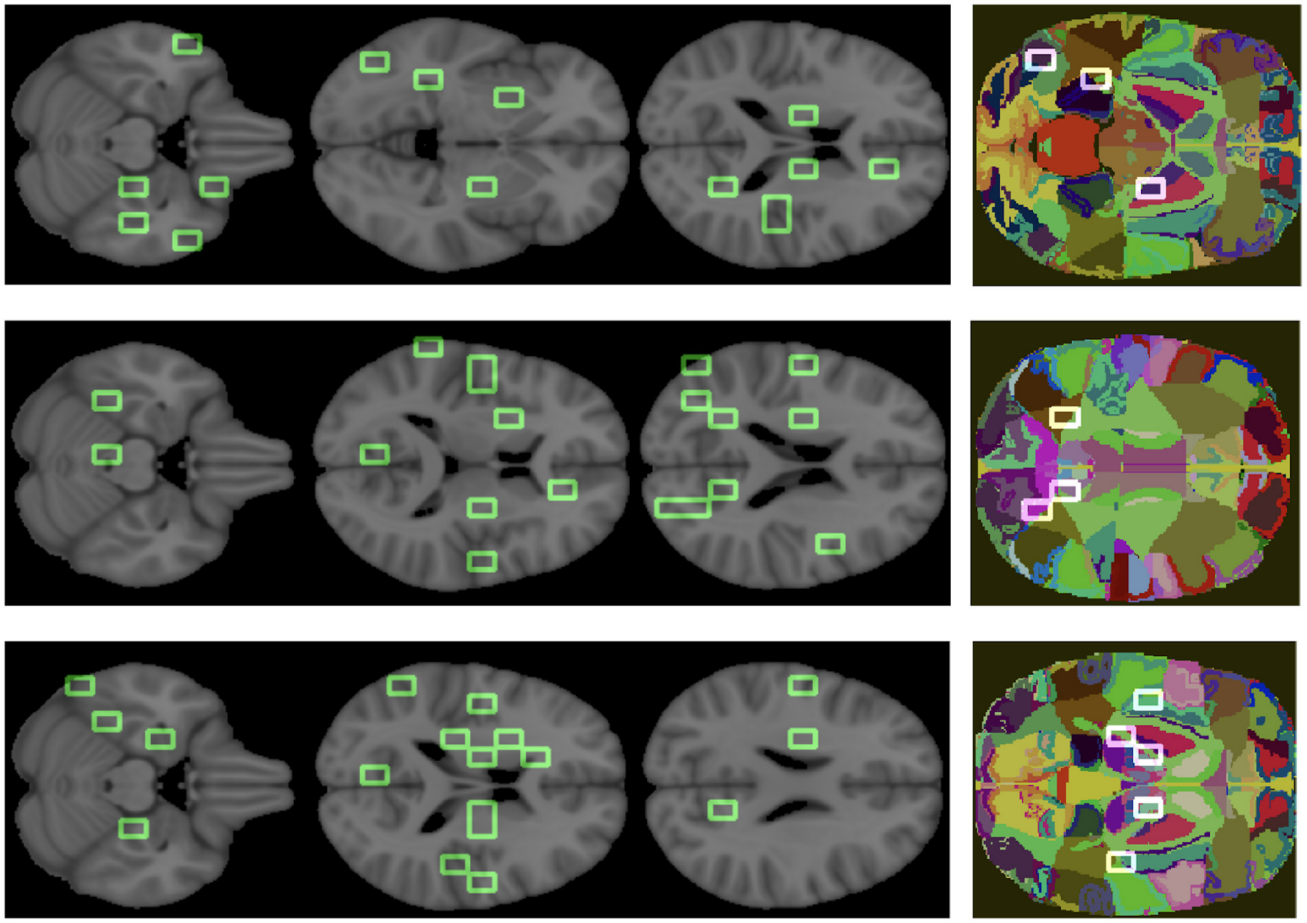
Important features are mapped over the brain. The three colored images represent the overlapping of some patches, we obtained from the analysis, on the regions of the atlas. From top to bottom, the graphs correspond to AD, MCI, NC.

Finally, to clinically validate the methodology we associated an anatomical district to each important feature (see Figure 8).

The goal was to show the model consistency by demonstrating that the outlined regions are clinically related to AD. For what concerns the overall ranking, the brain regions found were putamen, middle and superior temporal gyrus, anterior cingulate, precentral and postcentral gyrus, insula, sub-gyral, thalamus, culmen, lingual gyrus, cuneus, middle occipital gyrus, and brain stem. Analogously, we found the most relevant brain regions for the class-specific rankings.

There are a number of brain regions that were included in the best 30 for AD, including the amygdala, parahippocampal gyrus, fusiform gyrus, precuneus, posterior cingulate, as well as other regions previously ranked in global rankings (insula and lingual gyrus) that represent additional features nodes. For the MCI class, the following brain regions have been included in the top 30: posterior cingulate, precuneus and others already found in the global ranking, such as middle temporal gyrus, sub-gyral, and insula. The following regions are ranked in the top 30 for the NC class: uncus, parahippocampal gyrus, lateral ventricle, caudate, precuneus, posterior cingulate, and other regions already found in the global ranking (culmen, thalamus).

## 4. Discussion

The proposed approach aimed at evaluating brain connectivity as a tool for Alzheimer's disease staging and its explainability. We set up a three-label classification problem (NC, MCI, AD) in order to estimate whether the brain connectivity model, based on T1 brain MRI, was able to accurately distinguish these clinical conditions and whether the SHAP could offer support to explain the decision-making of a classifier. Our findings perfectly match performances presented in international challenges (Bron et al., 2015; Amoroso et al., 2018a; Dimitriadis et al., 2018; Sørensen et al., 2018; Lin et al., 2021); the proposed connectivity model can suitably characterize the disease onset and the anticipating cognitive impairment.

We compared several classification models to ensure that the method accuracy depended on the brain connectivity model proposed more than learning algorithms. In fact, we found accuracy levels algorithm independent. Among the different models, the most reliable classifier was RF which was able to achieve a three-class median accuracy of 66% followed by SVM with practically indistinguishable performance. Further, we verified that these models were also in agreement from the prediction point of view, in fact the classification scores achieved a Pearson's correlation >0.70 for all three classes.

A large number of studies have achieved impressive results related to AD/NC binary classification in recent years. Using machine learning techniques, the accuracy value could vary between 80 and 90% (Amoroso et al., 2018b; Gupta et al., 2019; Sheng et al., 2021; Zhao et al., 2021). Although excellent, these binary results do not take into consideration the most interesting clinical class, MCI subjects. As a result of the heterogeneity of this intermediate stage, studying MCI patients is quite challenging, but also complex. Three-label classification makes the study much more complex, which explains why accuracy performance is not excessively high compared to binary classifications (Cabral and Silveira, 2013; Sørensen et al., 2014; Cárdenas-Peña et al., 2016; Lama et al., 2017; Lee et al., 2019; Jimenez-Mesa et al., 2020).

Among the five classifiers considered here, the worst results were obtained by NB and LR classifiers. This result could be due to high-dimensional feature space; RF and SVM are more flexible to handle dataset with a high number of features (Badillo et al., 2020; Myszczynska et al., 2020; Spooner et al., 2020). A further factor that could have affected these results is the small sample size, which could certainly have represented a limitation to the performance of the classifiers. The difficulty of finding many valid clinical data, especially in the field of neuroimaging, makes it necessary to use high dimensional data with small number of samples and improve the models on these same small datasets (Vabalas et al., 2019). Combining this limit with the need to use many features can make the training sample affected by dataset blind spots, producing model performances that are highly variable compared to the real ones (Berisha et al., 2021). This result suggests the importance of considering algorithms like RF or SVM and, therefore, carrying out explainability analyses to make these models more interpretable for both patients and clinical practitioners. Thus, we investigated the influence of different features on classification using the XAI approach based on SHAP. By using this methodology, we can gain a better understanding of how algorithms work and how artificial intelligence methods can be used in clinical practice. As a means of evaluating how features affect RF classifier predictions, we conducted a global analysis that included all subjects in the dataset and compared it with the three class-specific analyses that included only patients correctly classified. We found a significant agreement between global feature importance and the class-specific one of MCI and NC classes, while the association tends to be weaker for the AD ranking. This result confirms how AD brain atrophy affects connectivity in highly heterogeneous ways (Zhang et al., 2016; John et al., 2017; Poulakis et al., 2018; Sui et al., 2018; Badhwar et al., 2020). Moreover, by comparing the three class-specific rankings we found no significant correlation; these findings emphasize again the disease heterogeneity and show how brain connectivity within each class follows substantially different patterns (John et al., 2017; Khazaee et al., 2017; Yu et al., 2017; Sheng et al., 2019).

Since the SHAP method provides information for analyzing personalized predictions about individual patients, we also presented some personalized analyses as an example. This is particularly important to envisage future developments toward personalized diagnostics and treatment (Fellous et al., 2019; Van der Velden et al., 2022; Vrahatis et al., 2023). Moreover, this is also important as our findings confirm that class-specific rankings can significantly differ from global importance: it is not possible to conceive a subject-specific model of AD without considering the subject's peculiarity. Finally, explainability analysis also provides a way clinically validate the proposed brain connectivity model in that it allows to directly relate the features driving classification to specific brain regions. In particular, our findings demonstrate that connectivity metrics reveal brain regions, such as parahippocampal gyrus, amygdala, uncus, fusiform gyrus and lateral ventricle, whose relation with AD is established (Pearson et al., 1985; Scahill et al., 2002; Zhang and Wang, 2015; Amoroso et al., 2018b).

## 5. Conclusion

In this work, a structural brain connectivity model was proposed to study Alzheimer's disease and mild cognitive impairment; to this aim an accurate three-label classification model was designed and, based on its decision scores, an explainable SHAP approach was implemented. To the best of our knowledge, this is the first attempt to equip a novel patch-based connectivity model with an XAI framework. Moreover, to ease interpretability, SHAP were mapped onto a brain atlas; the main advantage of this method, in fact, is that it allows to directly relate mathematical graph entities to anatomical districts. The accuracy and the robustness of the network model were assessed by comparing several classifiers. XAI analyses provided additional information about both global and patient-level explanations; in particular our findings confirmed that despite the presence of well determined regions related to AD, each patient deserves specific attention in that the disease heterogeneity makes its patterns extremely varying. Future studies could investigate how this variability reflects the specific brain resilience to the disease and, therefore, the possibility to design stage-specific therapies. Moreover, while here the wide MCI class was considered, further studies could also the investigate differences within this class. The proposed approach is general and it could be hopefully applied to shed light over several pathologies; besides, its interpretability could ease its adoption in a domain where learning algorithms are mistrustfully seen as black boxes.

## Data availability statement

The original contributions presented in the study are included in the article/Supplementary material, further inquiries can be directed to the corresponding author.

## Ethics statement

All experiments were performed with the informed consent of each participant or caregiver in line with the Code of Ethics of the World Medical Association (Declaration of Helsinki). Local institutional Ethics Committees approved the study.

## Author contributions

NA designed the experiments. SQ and ML carried out the analyses. NA and SQ wrote the original draft. AM and RB supervised the analyses. All authors revised and approved the manuscript.
