# Supplementary material for: An eXplainability Artificial Intelligence approach to brain connectivity in Alzheimer's disease
Source: Front Aging Neurosci. 2023 Aug 31;15:1238065. doi: 10.3389/fnagi.2023.1238065 (PMC10501457; doi:10.3389/fnagi.2023.1238065)
Supplement: Supplementary file 1 [file Data_Sheet_1.pdf]

Supplementary Material

1 SUPPLEMENTARY TABLES AND FIGURES

1.1 Figures

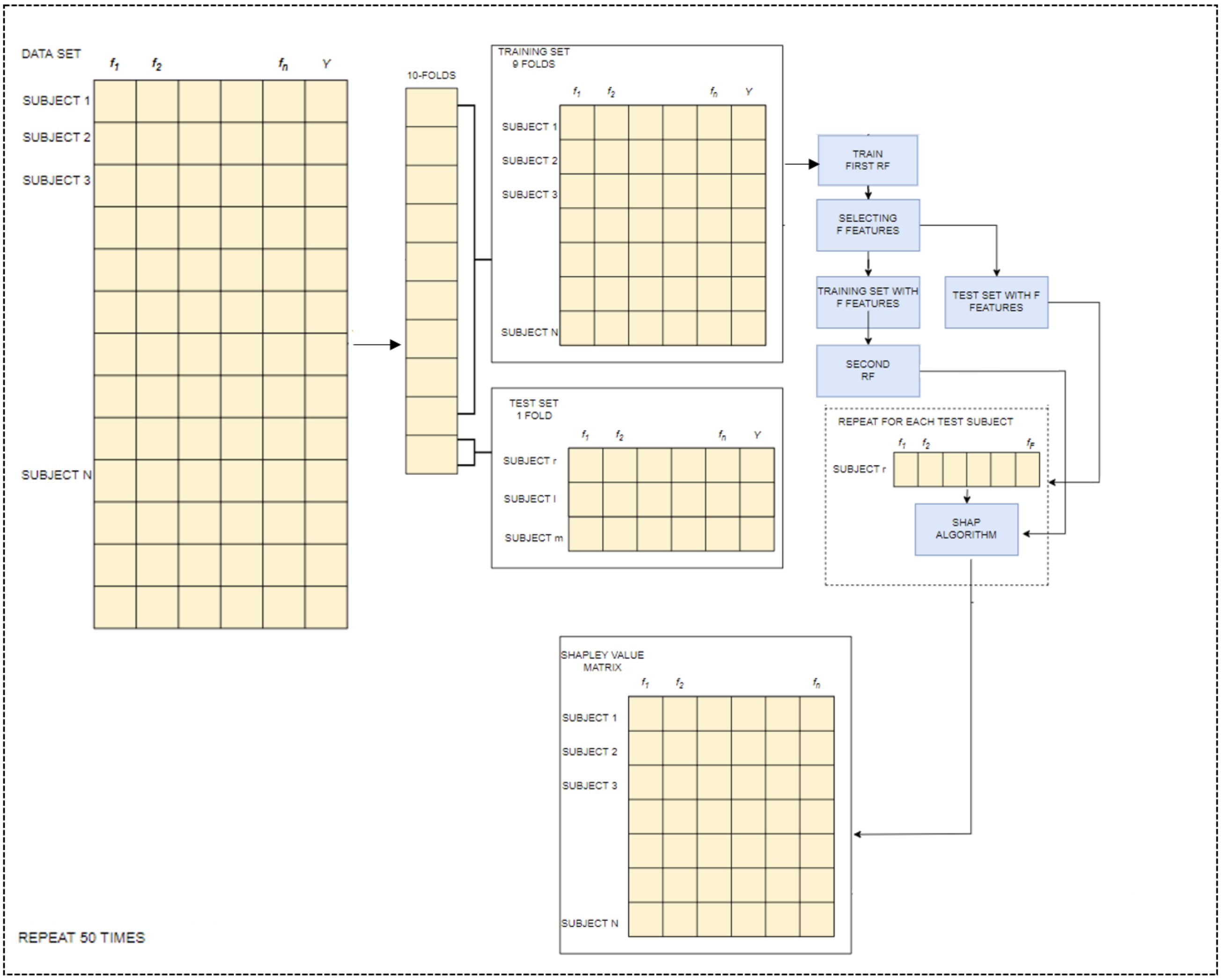

Figure S1. Workflow of the proposed XAI model.

1.2 Tables

The following Table S1 shows accuracy, balanced accuracy, specificity, sensitivity and precision obtained using the RF as classifier.

	AD	MCI	NC	Total
Accuracy	0.84 ± 0.05	0.69 ± 0.07	0.78 ± 0.07	0.66 ± 0.07
Balanced Accuracy	0.70 ± 0.08	0.69 ± 0.07	0.74 ± 0.08	0.71 ± 0.06
Specificity	0.94 ± 0.04	0.68 ± 0.1	0.82 ± 0.08	0.81 ± 0.04
Sensitivity	0.46 ± 0.16	0.73 ± 0.1	0.66 ± 0.14	0.62 ± 0.08
Precision	0.71 ± 0.18	0.68 ± 0.07	0.62 ± 0.12	0.67 ± 0.09

Table S1. Performance metrics of the RF model.
